# Enuresis in Children

**Published:** 1904-02-06

**Authors:** 


### ENURESIS IN CHILDREN.

In suggesting a rational line of treatment for this oftentimes extremely troublesome complaint, Dr. Percy Lewis[1] points out that the subjects of the malady are mostly anæmic and unhealthy in aspect. They generally are not fond of meat, eat irregularly, and feed chiefly on starchy and sugary foods. They are disinclined for either mental or physical exertion. If such children are roused during the night to pass water, they are awakened with difficulty, and at once relapse into slumber on being put back to bed. In quite a short time afterwards it may be discovered that they have wetted the bed. If the urine be measured in these cases it will be found that the amount passed in a night is seldom less than two pints and it may be as much as four pints. On analysis the urine shows a very low specific gravity, a neutral or alkaline reaction and perhaps a deposit of triple phosphates or oxalates. Frequently a trace of albumen is present. The polyuria disappears in the daytime except in cases when the incontinence is continued during the day. On the basis of these observations Dr. Lewis recommends reducing the carbohydrates in the food. He finds that in most cases a rigid anti-diabetic diet removes the symptoms in a few days. The general weakness which has been produced by the excess of starchy foods must at the same time be treated with general tonics. During the process of cure, starchy foods may as a rule be allowed for breakfast. Without any other treatment hospital cases are relieved often at once, and finally cured, by being taken as in-patients and fed on the ordinary hospital diet. In private cases even small quantities of bread or cake, given at dinner or tea early in the treatment, cause the bed-wettings to recur. In about three to four weeks a normal diet may be usually given without enuresis occurring.

[1] Brit. Jour. of Children's Diseases, Jan. 1904.

